# The Left Atrial Appendage Revised

**DOI:** 10.21470/1678-9741-2017-0085

**Published:** 2017

**Authors:** Paulo Roberto Barbosa Evora, Antonio Carlos Menardi, Andrea Carla Celotto, Agnes Afrodite S. Albuquerque, Hannah Miranda Araujo Chagas, Alfredo José Rodrigues

**Affiliations:** 1 Division of Cardiovascular and Thoracic Surgery, Department of Surgery and Anatomy of the Faculdade de Medicina de Ribeirão Preto da Universidade de São Paulo (FMRP-USP), SP, Brazil.

**Keywords:** Atrial Appendage/Surgery, Heart Atria, Atrial Fibrillation, Cardiac Surgical Procedures

## Abstract

Nonvalvular atrial fibrillation is associated with a 4- to 5-fold strokes increase and may be responsible for 15% to 20% of all strokes in the elderly. In this scenario, the left atrial appendage thrombus would be the associated with 90% of cases. The use of anticoagulants, percutaneous devices, and the left atrial appendage surgical exclusion is still an open discussion. For left atrial appendage procedures, relevant anatomic spatial relationships have to be emphasized, besides the chance of the normal physiological functioning would be eliminated with the proceedings. There are evidences that the left atrial appendage closure during routine cardiac surgery is significantly associated with an increased risk of early postoperative atrial fibrillation. Therefore, the purpose of this review is to focus basic aspects for continuous medical education. In summary, the rationale of this text is to emphasize anatomical and pharmacological aspects involved in the simple surgical exclusion of left atrial appendage under cardiopulmonary bypass. There are several operative techniques, but to conclude this revision it will present one of them based on the discussed basic sciences.

**Table:** 

Abbreviations, acronyms & symbols	
AF	= Atrial fibrillation
CPB	= Cardiopulmonary bypass
ED	= Endothelial dysfunction
EE	= Endocardial endothelium
EECs	= Endocardial endothelial cells
LA	= Left atrium
LAA	= Left atrial appendage
NO	= Nitric oxide
POAF	= Preoperative atrial fibrillation
RAS	= Renin-angiotensin system
TEE	= Transesophageal echocardiogram

## INTRODUCTION

Nonvalvular atrial fibrillation (AF) is associated with a 4- to 5-fold strokes increase and may be responsible for 15% to 20% of all strokes in the elderly. In this scenario, the left atrial appendage (LAA) thrombus would be associated with 90% of cases, even considering the effective oral anticoagulants, they may be used only in 40% to 50% of patients at stroke increased risk. Nowadays, the Watchman is the only device approved in the United States. However, this approach includes at least three unresolved issues: 1) Optimal patient selection criteria; 2) The devices role in patients in whom anticoagulation is contraindicated; and 3) The novel oral anticoagulants role *versus* the device not tested in randomized trials^[1]^. The left auricle occlusion is adopted by all the current guidelines for the prevention of thromboembolism and stroke, motivating the development of various percutaneous devices occluders, and has been practiced since the 1930s^[2]^, even without evidence-based data in the form of randomized controlled trials. Initially, LAA closure through excision or ligation was performed in the context of open heart surgery or even during abdominal surgeries. There are evidences that the LAA closure during routine cardiac surgery is significantly associated with an increased risk of early postoperative AF. However, remains uncertain whether the prophylactic exclusion is warranted for stroke prevention during cardiac surgery. There are evidences that the LAA closure during routine cardiac surgery is significantly associated with an increased risk of early postoperative AF. Therefore, the purpose of this review is to focus basic aspects for continuous medical education. In summary, the rationale of this text is to emphasize anatomical and pharmacological aspects involved in the simple surgical exclusion of LAA under cardiopulmonary bypass (CPB). There are several operative techniques, but to conclude this revision it will present one of them based on the discussed basic sciences, without considerer "stapling" devices which can be applied from the outside surface of the heart.

For the review of the general aspects of the subject, the original text selected was published by Holmes and Reddy^[1]^.

## LEFT ATRIAL APPENDAGE ANATOMY

The publication chosen for the description of the LAA anatomy was signed by DeSimone et al.^[3]^ from Mayo Clinic, Rochester, MN, USA. The three-dimensional LAA morphology is, by itself, the substrate for thrombus generation, and should be the subtract for embolism due to its direct connection to the left-sided circulation. The LAA mesodermal justify its exclusion from the atrial circulation and thereby can lead to a significant reduction in stroke risk. This process also provides insight into the LAA as an endocrine organ, its fluid homeostasis involvement, and its autonomic nervous system connection. The surrounding LAA structural knowledge arrangement is critical to identify the endocardial and epicardial landmarks perspective to improve devices placement. Furthermore, correlation of the LAA body, neck, and ostium to the surrounding anatomy can also improve both procedural safety and efficacy. Also, a working knowledge of the regional anatomy adds a prudent degree of awareness for procedural complications allowing for early identification and timely intervention. A detailed understanding of the LAA morphology (embryology, histology, and gross anatomy) is imperative to identify the individual approach for each patient^[3]^.

Cardiac structures are derived from the mesodermal layer during the third week of embryonal development, when LAA originates and develops from the left side of the primary left atrium. At around week six of embryologic life, further development of the left atrium occurs around the six weeks, depending on the pulmonary system growth and development, which connects to the heart via the pulmonary vein-left sinus horn^[4,5]^. The LAA is derived from the primary atrium left the wall, during the fourth week of embryonic development, when has ultrastructural, and physiological characteristics distinct from the left atrium^[6]^.

The appendage is comprised of rigid pectinate muscles, in contrast to the rest of the smooth left atrium, with thin-walled myocardium interdigitating these raised regions. These pectinate structures are almost exclusively found in the LAA, in comparison to the remainder of the left atrium, and these anatomical variations can influence the exclusion procedures deserving to be reviewed: 1) The LAA pectinate muscles; 2) The LAA variable morphology of its ostium shape and dimensions; and 3) The LAA variable morphology and stroke risk^[3,7]^. All these factors have been extensively studied resulting in great controversies.

Relevant LAA anatomic spatial relationships have to be emphasized since it is a blind-ended pouch situated within the pericardium emerging from the left atria. The anatomical relations are complex, and the critical structures surrounding the LAA include: 1) The pulmonary artery superiorly directed; 2) The appendage tip pointing inferomedialy oriented towards the left ventricle free wall; 3) The left phrenic nerve running overtop of the appendage; 4) Fibers of Bachmann's bundle that approach the LAA from the medial aspect of the atrial roof; 5) A posteriorly situated left superior pulmonary vein; and 6) An inferiorly related mitral valve^[3]^. However, of utmost importance to note, when performing any LAA procedure, it is its critical relationship with the area above the left atrioventricular groove, which houses both the left circumflex artery and great cardiac vein (Table 1).

**Table 1:** Left atrial appendage anatomic relationships.

Relationship of the left circumflex artery to the LAA ostium
Epicardial/endocardial relationship of the LAA to the left superior vena cava and left superior pulmonary vein
Relationship of the LAA ostium and mitral valve
Relationship of the left circumflex artery to the LAA ostium
Epicardial/endocardial relationship of the LAA to the left superior vena cava and left superior pulmonary vein
Relationship of the LAA ostium and mitral valve

LAA=left atrial appendage

## LEFT ATRIAL APPENDAGE PHARMACOLOGY

The endocardial endothelium (EE) is a monolayer cellular that covers internally the heart. The endocardial endothelial cells (EECs) also constitute a very large contact surface area that offers a very high ratio of cavity surface area to atrial volume, a finding that suggests an important sensory role for the EE. These cells play the role of a physic-chemical barrier between the cardiomyocytes and the circulating blood^[7-9]^. Furthermore, just like other types of endothelial cells, EECs release several factors such as nitric oxide (NO), angiotensin II^[7]^, endothelin and prostacyclin^[8-10]^.

**Fig. 1:**
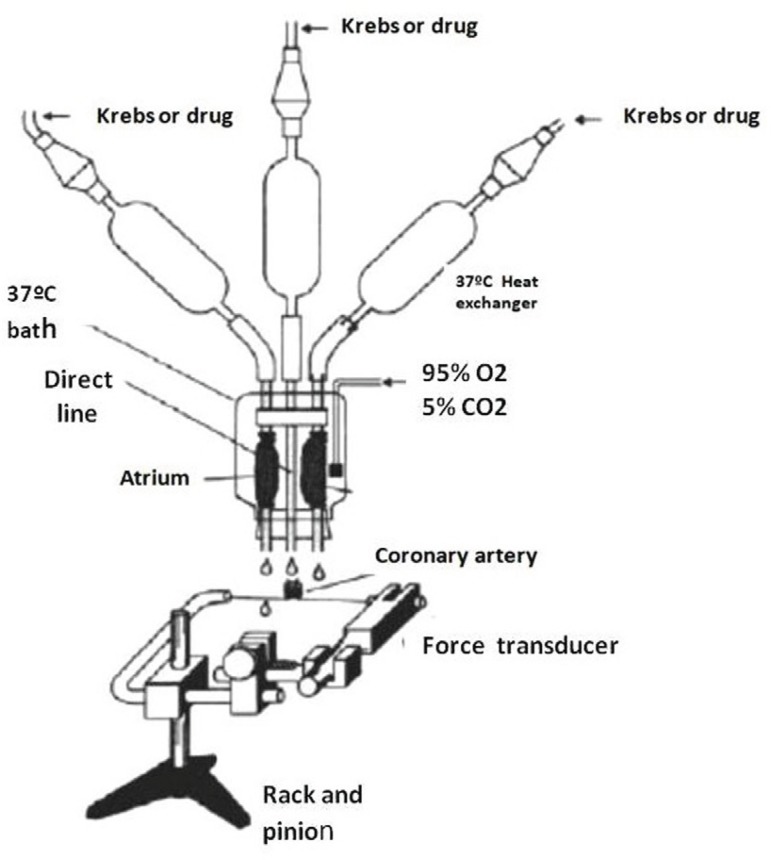
Bioassay system from the perfused atrial appendage tube. The atriums are perfused through a separate constant-flow perfusion loop, and vasoactivity of effluent from the atriums was bioassayed on a ring of canine coronary artery. The central cannula was used to direct perfusion of coronary rings (physiologic solution with or without drugs). The lateral cannulae were connected to the right and left atrium and the solution perfused thought atrial tubes was dripped on coronary ring. The coronary ring was connected to a force transducer that registers the variation of vascular tone (contraction and relaxation)^[10]^.

**Fig. 2:**
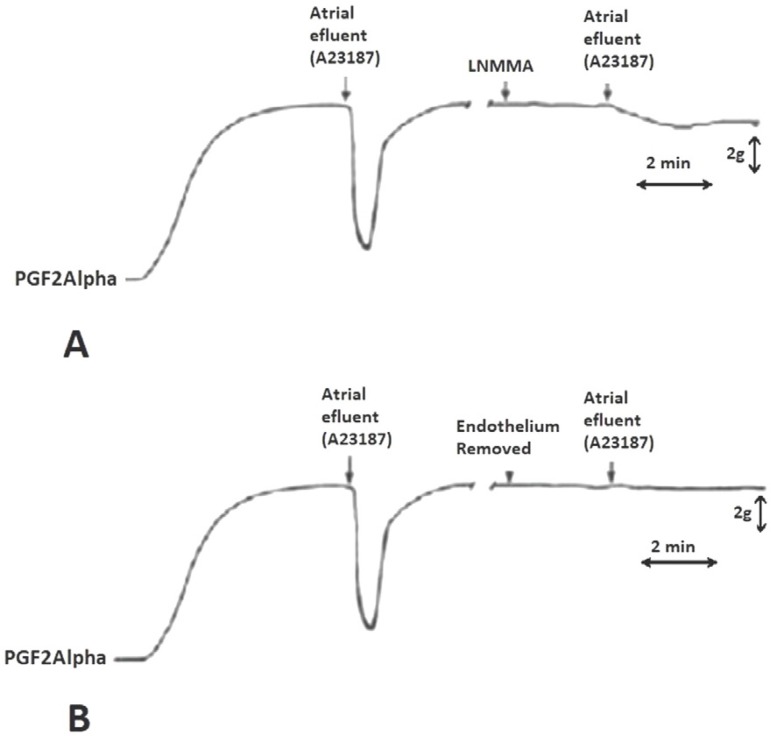
Representative recording of change in contraction of canine coronary artery ring with endothelium superfused with effluent from atrial appendage tubes infused with A23187. Relaxation was inhibited when the ring was superfused with L-NMMA (A) and when the endothelium was removed (B) ^[10]^.

**Fig. 3:**
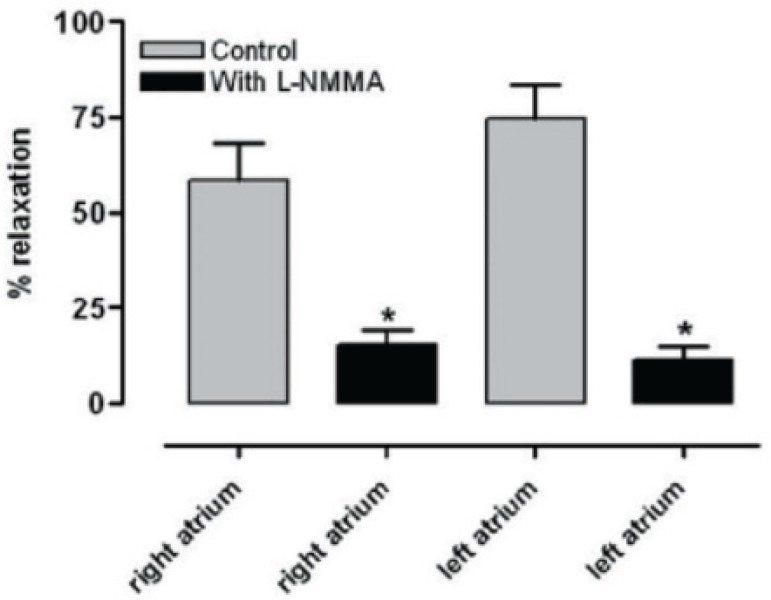
Bioassay of calcium ionophore-induced promote relaxation in the right and left atrium. The vasodilator activity of effluent from the right and left atrial appendage was blocked 85.1 ± 7.9% and 88.9±3.5%, respectively, by the presence of the L-NMMA (10-M) in rings contracted with PGF 2 (2x10 M). Results are reported as means±SEM. * indicate significant difference between with and without L-NMMA groups (P<0.001)^[10]^.

To study the NO release from intact atrial endocardial endothelium, tube-shaped sutures of canine atrial appendages were performed and effluents from these tubes were bioassayed (isolated perfused organ chamber system) for detection of NO in the canine coronary artery (Figure 1). Effluent from the right atrial appendage caused a relaxation of 58.4 ± 10.1% and the left atrial appendage 74.9 ± 8.5% from the initial prostaglandin-F2α contraction in the bioassayed coronary artery (Figure 2). This relaxation was abolished by treating the heart tubes with Triton X-100 and reduced by treatment with L-NMMA, a competitive inhibitor of NO and with indomethacin, an inhibitor of the cyclooxygenase pathway also indicating the release of vasodilatory prostanoids from the endocardial endothelium (Figure 3). This study showed, for the first time, *in vitro* luminal release of NO and prostacyclin from the canine heart atrium. The ability of the EE to produce these factors could play an important role in preventing thrombus formation in the cardiac chambers. In the present model, it could not demonstrate a basal release of EDNO (*i.e.* release in the absence of stimulation by an agonist from the intact atrium, as is typically present in other vessels^[10]^.

Guazzi and Arena^[11]^ focuses on the evidence of an association of AF with endothelial dysfunction (ED) that increases proinflammatory agents, oxidative stress and impairs NO-dependent vasorelaxation. There are some evidences that AF is a risk factor for ED: 1) Reduced plasma nitrite/nitrate levels impaired; 2) Increased blood flow acetylcholine-mediated; 3) Impairment of flow-mediated dilatation by comorbidities; and 4) Efficacy of cardioversion. Speculative mechanisms sustain the AF-ED association: 1) An impaired rheology associated with AF induced by a non-pulsatile turbulent flow, which may impair the NO synthase (eNOS) activation; 2) A disorganized atrial contraction markedly reduces eNOS expression; 3) AF causes a proinflammatory activity with elevation of C-reactive protein and cytokines; and 4) Systemic factors such as the renin-angiotensin system (RAS) may be prominent in a kind of inflammation reciprocally "cross-talk" and the RAS inhibition prevents AF^[11]^.

## THE CONTROVERSY ABOUT LEFT ATRIUM APPENDAGE FUNCTION

The LAA role as an endocrine organ is greatly underappreciated^[12]^. The LAA contain a variety of cardiac progenitor cells that is fundamental to many of its underlying functions, including its vital role in endocrine regulation^[13]^. The LAA has critical importance to homeostasis and cardiovascular physiology, containing almost 30% of the heart atrial natriuretic factor, which is a potent endocrine modulator including a change in heart rate, natriuresis, and urination, effects suggestive of the role in modulating body volume status. Therefore, there was an old controversy about the LAA critical role in a healthy physiological function that may be eliminated with occlusion procedures, for example, to keep the critical pressure-induced, stretch receptor endocrine response^[14]^. However, the right atrial appendage can execute similar LAA endocrine effects overcoming this dilemma. Otherwise, the endocrine regulation should need both LAA structures, and the LAA exclusion may eventually lead to long-term adverse results, but no studies to date have shown any adverse effects post-LAA exclusion^[12-14]^.

## LEFT ATRIAL APPENDAGE AND STROKE

The observation that 90% of the thrombi found in nonvalvular AF patients and 57% found in valvular AF are in the LAA triggered significant interest in the LAA as a potential therapeutic target. Until recently, the results were inconsistent, and high rates of incomplete occlusions precluded the medical community from confirming a definite relationship between LAA and stroke. Anticoagulation is considered the first-line for stroke risk reduction in AF, and the American College of Cardiology/American Heart Association guidelines recommend LAA exclusion only with surgical ablation of AF or in the context of concomitant mitral valve surgery^[15]^. Nowadays, considering the occlusion devices development Ramlawi et al.^[15]^ recommend LAA exclusion in all AF patients undergoing cardiac surgery regardless of their suitability for anticoagulation therapy. For lone AF patients with an embolic risk that necessitates anticoagulation, it was recommended LAA exclusion in those who have failed or with relative or absolute contraindications to anticoagulation. Characteristics of the ideal LAA exclusion include: 1) The procedure is safe and minimally invasive; 2) The procedure is complete and receives intraoperative transesophageal echocardiogram (TEE) confirmation; 3) The procedure is free of intracardiac foreign bodies; 4) The procedure is applicable to all LAA morphologies; 5) The procedure allows the immediate cessation of anticoagulation medications; and 6) The cost is lower compared to other devices and long-term oral anticoagulation^[15]^.

## ROUTINE SURGICAL EXCLUSION OF THE LEFT APPENDAGE

Although the LAA occlusion is technically easy, its regular performance is still a matter of open discussion. A recent study from Mayo Clinic presented in the Scientific Bulletin of the Brazilian Society of Cardiovascular Surgery (number 01/2017) concluded that there was no need for LAA exclusion and, surprisingly, associated this surgical maneuver with a higher incidence of postoperative AF^[16]^. The study included 9,792 adults who underwent CABG or valve surgery between January 2000 and December 2005, matching a propensity-score analysis. Twenty-eight covariates pretreatment were performed, 461 matching pairs were derived and analyzed to estimate the association of LAA closure with early postoperative AF (AF ≤ 30 days of surgery), ischemic stroke and mortality. The authors concluded that after adjustment for treatment bias, LAA closure during routine cardiac surgery was significantly associated with early preoperative AF (POAF) increased risk, but without any risk of stroke or mortality. It remains uncertain whether prophylactic exclusion of LAA is warranted for stroke prevention during non-AF-related cardiac surgery. Therefore, the decision of "to close or not to close" the appendage is an open question and therefore depends on the individual experience of the surgeon^[16]^.

## SURGICAL ASPECTS - "HOW TO CLOSE"

Some surgeons, including the authors of the present review, routinely close the LAA in patients having mitral valve surgery, without any adverse results, in accordance with the literature data. Studies suggest that the LAA has a minimal useful function, and it is the source of most embolism causing hundreds of thousands of strokes annually. It is clear that the LAA is the most human lethal appendage. During cardiac surgery, the LAA removal is safe and has to be considered^[17]^. According to the 2017 Society of Thoracic Surgeons Clinical Practice Guidelines, the surgical ablation for AF can be carried out without major morbidity or additional risk of operative mortality, and is recommended at the time of joint mitral operations, at the time of isolated aortic valve replacement, isolated coronary artery bypass graft surgery, and concomitant aortic valve replacement plus coronary artery revascularization^[18]^. There is an association between LAA decreasing velocity, measured by TEE, and the development of postoperative AF^[19]^. Also, the LAA reduction flow velocity is a risk factor for thrombus formation and increases the risk of stroke in patients with AF. Furthermore, in patients with AF, LAA orifice diameter and LAA volume, but not left atrial dysfunction, were determinants of stroke and were useful for stratifying noncardioembolic risk in patients with AF^[20]^. Therefore, these two pieces of evidence (LAA decreasing flow and volume) reinforce the surgical LAA exclusion.

## TECHNIQUE

**Fig. 4:**
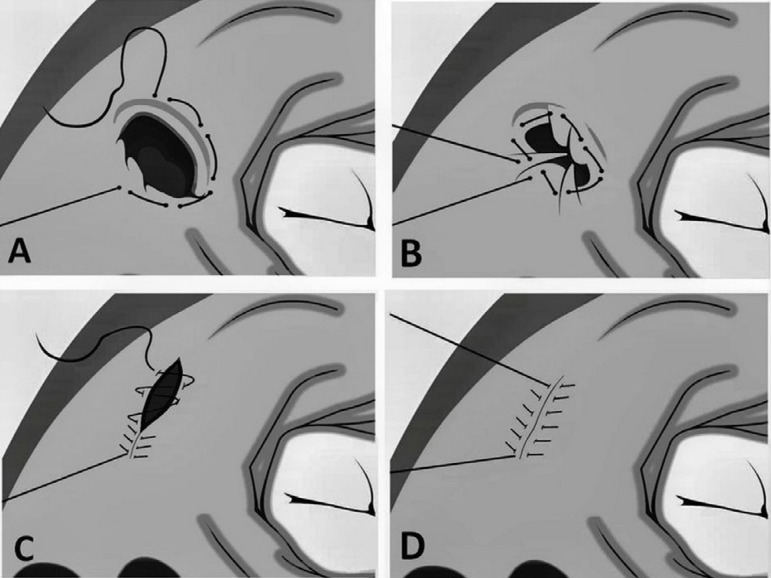
Surgical technique. A - Purse-string suture avoiding the left atrial appendage; B - The purse-string suture is tied up taking care not to be overtight, because the aim is to delineate the rims, not to obliterate the orifice completely; C - Finally, a second "out-out" running suture carried out and then tied up; D - Final aspect.

Assuming "to close" LAA to prevent thromboembolism, we will present, as illustration, a technique based on the discussed basic concepts. The LAA should be damaged seriously, which explains the historical reluctance to manipulate appendage based on its fragility and proximity of surrounding structures. Unsuccessful results, regardless of the technique employed, have been reported. An incomplete LAA occlusion has been reported in 10% to 73% of patients, depending on simple running or double suture. Although excision seemed more practical (success rate of 73%), it was observed a residual stump in 27% of them^[20,21]^. Our preference, used since the last 20 years in the association of mitral valve surgery, is quite similar to the technique described by Hernandez-Estefania et al.^[22]^. After entering the left atrium (LA) by the atrioventricular groove, the LAA is completely invaginated, taking care in avoiding lesion to the LAA apex. A 4-0 polypropylene purse-string suture is placed along the base of the appendage including only atrial tissue (remember the LAA anatomical individuality) (Figure 4A). The stitches should be enough for encircling the shape inlet orifice avoiding the LAA (remember the LAA embryological individuality). To pass across the intimal and medial tears, avoiding transfixing, is a critical maneuver. While LAA is pulled outward with forceps, the two ends of the suture are pulled together gently, and the purse-string suture is tied up. The purse string suture has not to be overtight because the aim is to delineate the rims, not to obliterate the orifice completely (Figure 4B). Finally, a second "out-out" running suture carried out and then tied up (Figures 4C and 4D). It is expected that the right atrial appendage will maintain the pharmacological functions of the excluded LAA.

## CONCLUSION

The mini-review concluding remarks would be summarized in Table 2.

**Table 2:** Concluding remarks.

Patients with nonvalvular AF have a 4- to 5-fold increase in strokes and that rhythm may be responsible for 15% to 20% of all strokes, particularly in the elderly.
Unresolved issues include 1) Optimal patient selection criteria; 2) The role of devices in patients in whom anticoagulation is contraindicated, and; 3) The relative role of novel oral anticoagulants *versus* the device which has not been tested in randomized trials.
For LAA procedures relevant anatomic spatial relationships have to be emphasized.
There was the early controversy that the critical role of the LAA in normal physiological functioning may be eliminated with LAA procedures.
LAA closure during routine cardiac surgery was significantly associated with an increased risk of early POAF but did not influence the risk of stroke or mortality. It remains uncertain whether prophylactic exclusion of LAA is warranted for stroke prevention during non-AF-related cardiac surgery.

AF=atrial fibrillation; LAA=left atrial appendage; POAF=preoperative atrial fibrillation

**Table:** 

Authors' roles & responsibilities	
PRBE	Conception and study design; analysis and/or data interpretation; manuscript writing or critical review of its content; final manuscript approval
ACM	Conception and study design; analysis and/or data interpretation; critical review and final manuscript approval
ACC	Conception and study design; analysis and/or data interpretation; critical review and final manuscript approval
AASA	Conception and study design; analysis and/or data interpretation; critical review and final manuscript approval
HMAC	Conception and study design; analysis and/or data interpretation; critical review and final manuscript approval
AJR	Conception and study design; analysis and/or data interpretation; critical review and final manuscript approval
